# Association between dietary mineral nutrient intake, body mass index, and waist circumference in U.S. adults using quantile regression analysis NHANES 2007–2014

**DOI:** 10.7717/peerj.9127

**Published:** 2020-05-04

**Authors:** Shan Jiang, Xiaoyu Ma, Meng Li, Shoumeng Yan, Hantong Zhao, Yingan Pan, Changcong Wang, Yan Yao, Lina Jin, Bo Li

**Affiliations:** Department of Epidemiology and Biostatistics, Jilin University School of Public Health, Changchun, Jilin, People’s Republic of China

**Keywords:** Mineral elements, Body Mass Index, Waist circumference, Quantile regression

## Abstract

**Objective:**

Mineral nutrients play an important role in maintaining material and energy metabolism. Reports on mineral nutrient intakes and body mass index (BMI) and waist circumference (WC) are rare in the United States. This study examined the relationship between BMI, WC and dietary mineral intakes.

**Method:**

We used the data from National Health and Nutrition Examination Survey 2007–2014. Nutrient intakes were adjusted for energy according to the residual adjustment method. We used the quantile regression model to analyze the relationship between BMI, WC under different distributions and the average daily mineral intakes.

**Result:**

A total of 19,952 people were included in the study, including 9,879 men and 10,073 women (≥20 years old). The median BMI was 27.935 kg/m^2^ and the median WC was 97.700 cm. The results of quantile regression showed that calcium, magnesium, potassium, copper, zinc and iron intakes were negatively correlated with BMI and WC, after adjusting for age and gender. Sodium and phosphorus intakes were positively correlated with BMI, sodium intakes were positively correlated with WC. This correlation was enhanced with increasing quantiles of risk levels. In high BMI or high WC populations, mineral intakes had a greater impact on BMI and WC. The quantile regression coefficients of selenium intakes were not statistically significant at each quantile.

**Conclusion:**

Our results suggested that the mineral nutrient intakes were associated with BMI and WC in American adults. However, we also need to further study the longitudinal effects of mineral intakes and obesity.

## Introduction

In 2015, excess weight contributed to 4.0 million deaths accounting for 7.2% of all causes of death (Afshin et al., 2017). Obesity can increase the incidence and mortality of chronic diseases such as diabetes and cardiovascular disease (Flegal et al., 2013). By 2016, more than 650 million adults have developed obesity worldwide (NCD Risk Factor Collaboration (NCD-RisC), 2017). The occurrence and development of obesity is a long-term process, and BMI and WC are sensitive indicators for the diagnosis of obesity (Caspard et al., 2018). Obesity has been a major public health problem worldwide.

In the past, most of the dietary measures for weight control focused on reducing the macronutrient intakes such as carbohydrates and fats. In recent years, more and more attention has been paid to the role of micronutrients in obesity. Epidemiological studies have shown that many people with obesity have inadequate intake of certain micronutrients, such as deficient in iron, calcium, magnesium, zinc and copper (Agarwal et al., 2015; Astrup & Bügel, 2019). Various studies suggested that there was a negative correlation between calcium intake and BMI, and that calcium intake can improve weight outcomes in overweight or obese individuals (Ilich et al., 2019; Lee & Cho, 2017; Rautiainen et al., 2016; Wadolowska et al., 2018; Zhu et al., 2013). A cross-sectional study in Poland showed that daily intake of minerals in postmenopausal females was related to BMI, overweight individuals have lower potassium and magnesium intakes, and higher sodium intake (Głąbska et al., 2016). In addition, cross-sectional studies in Mexico showed that specific nutrients have been associated with obesity, such as inadequate intake of iron, zinc (García et al., 2012, 2013). The systematic review indicated that obese individuals may have insufficient intake of antioxidants such as zinc, magnesium and selenium (Hosseini, Saedisomeolia & Allman-Farinelli, 2017). In Japanese schizophrenia, the intakes of phosphorus and salt were higher in overweight and obese individuals (Ito et al., 2015). South Korean National Health and Nutrition Examination Survey confirmed that high sodium intake may be a potential risk factor for weight gain independent of calorie intake (Yoon & Oh, 2013). High-dietary phosphorus, especially from foods processed with phosphate salts may be positively correlated with obesity (Anderson, 2013). Recent studies have shown that the mineral supplements can reduce body weight and inflammation and improve lipid metabolism, such as mineral supplements of calcium (Ilich et al., 2019), magnesium (De Baaij, Hoenderop & Bindels, 2015), zinc, copper and selenium (Khorsandi et al., 2019; Lee, 2018; Roman, Jitaru & Barbante, 2014).

However, there are few epidemiological studies of mineral intakes and obesity in the US. Therefore, the aim was to analyze the relationship between BMI, WC and mineral intakes in American adults. We particularly analyzed the impact of mineral intakes on low-weight and high-weight population to achieve early prevention and precision prevention.

## Method

### Study population

NHANES is a cross-sectional survey to assess the health and nutritional status of adults and children in the United States. It is a nationally representative sample of the non-institutional population in the United States. The NHANES database includes publicly available data released in 2 year cycles and is available from the NHANES website (http://www.cdc.gov/nchs/nhanes.htm). The data from four cycles of NHANES (2007–2008, 2009–2010, 2011–2012 and 2013–2014) were combined for the present analysis. A total of 40,617 individuals participated in the NHANES during 2007–2014, and our analysis was limited to 23,482 participants aged 20 years and over. Participants (*n* = 2,193) who lacked the data of BMI and WC were excluded. Of these, participants with incomplete or unreliable 24 h recall data (*n* = 1,080) were excluded. In addition, pregnant or lactating females (*n* = 247) were excluded. Extremely abnormal values of BMI, the largest five (>70 kg/m^2^) and the smallest five (<14.5 kg/m^2^), are removed. Finally, 19,952 participants (9,879 men and 10,073 women) were included in the analysis.

### Height, weight and waist circumference assessment

Weight, height and waist circumferences were measured in duplicate following standard procedures. Participants were weighed in light clothing using a digital scale with a precision of 0.1 g. Height of participants without shoes or hats was determined using a range finder with a 0.1 cm precision. BMI was calculated by dividing weight (kg) by height (m) squared. WC was measured with a 0.1 cm precision at the end of normal expiration. The horizontal position of the midpoint of the line connecting the lower edge of the costal arch and crista iliaca was taken as the measurement point.

### Dietary and supplemental intake assessment

USDA’s Food and Nutrient Database for Dietary Studies (FNDDS) 2007–2014 was used for processing the 2007–2014 intakes (http://www.ars.usda.gov/ba/bhnrc/fsrg). The FNDDS includes comprehensive information that can be used to code individual foods and portion sizes reported by participants and also includes nutrient values for calculating nutrient intakes. Because FNDDS is used to generate the nutrient intake data files for What We Eat in America, NHANES, it is not required to estimate nutrient intakes from the survey. FNDDS is made available for researchers to review the nutrient profiles for specific foods and beverages as well as their associated portions and recipes. Such detailed information makes it possible for researchers to conduct enhanced analysis of dietary intakes. FNDDS can also be used in other dietary research studies to determine the amounts of nutrients/food components in foods and beverages.

The data of dietary intakes included total nutrient intakes (Dietary Interview—Total Nutrient Intakes, First Day and Second Day) and dietary supplement intakes (Dietary Supplement Use 30 Day—Total Dietary Supplements). Total nutrient intakes were evaluated through a 24 h recall survey. The 24 h recall is a retrospective dietary assessment method, which provides the information of food intakes in the past 24 h. The first dietary interviews were conducted in the NHANES mobile examination center (MEC). The second dietary interviews were collected 3–10 days following the MEC dietary interview but not on the same day of the week as the MEC interview. If participants completed two 24 h recall surveys, we used the average dietary intake. Otherwise, we used a single and reliable 24 h recall. During the household interview survey, participants were asked what supplements, how often and how much they had taken in the past 30 days. The 30 day average dietary supplement intakes were used to assess the participants’ dietary supplement intake level. The total daily nutrient intakes were the sum of the nutrient intakes and the average daily intakes of the dietary supplement. According to the analysis guidelines provided by NHANES, the dietary weights were taken into account in all analyses. The examination protocol and data collection methods are fully documented in the NHANES dietary interviewers procedures manuals.

### Statistical analysis

We used R software (version 3.5.3; http://www.r-project.org) and IBM SPSS software (version 24.0) for statistical analysis. The mineral nutrients intakes were adjusted for total energy intake according to the residual adjustment method (Willett, Howe & Kushi, 1997). The distributions of dietary intakes, BMI and WC were determined to be non-normal according to the Kolmogorov Smirnov test (Table S1; Fig. S1). The median, maximum and minimum were used to represent the characteristics of continuous variables. The Mann–Whitney *U* test were performed to analyze the differences between men and women. The consumption of the various examined micronutrients is likely to be highly correlated. Therefore, the collinearity diagnostics of micronutrient intakes was made after the residual adjustment of the total energy. The ridge regression was carried out on the data to select the independent variables, because of the collinearity between the independent variables (Table S2). The quantile regression was performed to analyze the effects of the selected mineral nutrients on BMI and WC at different quantiles in the general population. For the non-normal distribution data, the quantile regression analysis is more suitable. Then, in order to ensure the full use of the information in the data, we performed the quantile regression analysis to analyze the effect of the removed independent variables on the dependent variables respectively. The confounding factors were adjusted in the quantile regression models such as age and gender. The *P* value less than 0.05 was considered statistically significant.

## Result

### Descriptive characteristics of participants

As shown in Table S1 and Fig. S1, dietary mineral intakes, BMI and WC were non-normally distributed. A total of 19,952 participants were included in the study, including 9,879 men (49.50%) and 10,073 women (50.50%). The median ages of men and women were 49 years. Table 1 showed the demographic information and dietary intake information of the participants. Among the participants, the median BMI was 27.935 kg/m^2^ and the median WC was 97.700 cm. Compared with men, women had significantly smaller WC (*P* < 0.001) and higher BMI (*P* < 0.001). Nutrient intakes were adjusted for energy according to the residual adjustment method. There were significant differences between men and women in the intakes of calcium, magnesium, copper, sodium, potassium, iron, phosphorus and selenium (*P* < 0.001), while there were no significant differences between men and women in zinc intakes.

**Table 1:** Characteristics of participants by gender (NHANES, 2007–2014).

	Total (*n* = 19,952)	Male (*n* = 9,879)	Female (*n* = 10,073)	*Z*	P*
	Median (min, max)	Median (min, max)	Median (min, max)		
Age/years	49.000 (20.000, 80.000)	49.000 (20.000, 80.000)	49.000 (20.000, 80.000)	−1.218	0.223
BMI (kg/m^2^)	27.935 (14.590, 69.000)	27.700 (14.590, 66.160)	29.220 (14.860, 69.000)	−5.581	<0.001
WC (cm)	97.700 (59.100, 176.000)	99.600 (61.800, 176.000)	95.500 (59.100, 172.500)	−17.514	<0.001
Calcium intake (g/d)^a^	1.371 (1.062, 7.762)	1.382 (1.062, 7.762)	1.362 (1.062, 5.300)	−3.674	<0.001
Magnesium intake (g/d)^a^	0.382 (0.317, 3.862)	0.387 (0.317, 3.862)	0.379 (0.317, 2.450)	−10.370	<0.001
Copper intake (mg/d)^a^	2.035 (1.552, 60.986)	2.050 (1.552, 60.986)	2.021 (1.552, 31.161)	−5.476	<0.001
Sodium intake (g/d)^a^	3.885 (3.391, 13.335)	3.962 (3.391, 12.464)	3.826 (3.391, 13.335)	−19.979	<0.001
Potassium intake (g/d)^a^	3.090 (2.627, 22.036)	3.139 (2.627, 22.036)	3.054 (2.627, 15.913)	−13.867	<0.001
Iron intake intake (g/d)^a^	0.025 (0.018, 0.253)	0.027 (0.018, 0.253)	0.024 (0.018, 0.220)	−22.085	<0.001
Phosphorus intake (g/d)^a^	1.517 (1.337, 6.142)	1.538 (1.337, 6.142)	1.500 (1.337, 3.093)	−15.956	<0.001
Selenium intake (mg/d)^a^	0.157 (0.129, 67.099)	0.159 (0.129, 3.470)	0.156 (0.129, 67.099)	−9.613	<0.001
Zinc intake (g/d)^a^	0.021 (0.015, 1.271)	0.021 (0.015, 1.271)	0.021 (0.015, 0.140)	−0.709	0.478
Energy (kcal/d)	1,910.000 (18.000, 13,509.000)	2,229.000 (162.500, 13,509.000)	1,664.500 (18.000, 9,595.000)	−52.312	<0.001

**Notes:**

a Nutrient intakes were adjusted for energy according to the residual adjustment method.

* Compared differences between men and women.

### Collinearity diagnostics and ridge regression analysis

**Figure 1:**
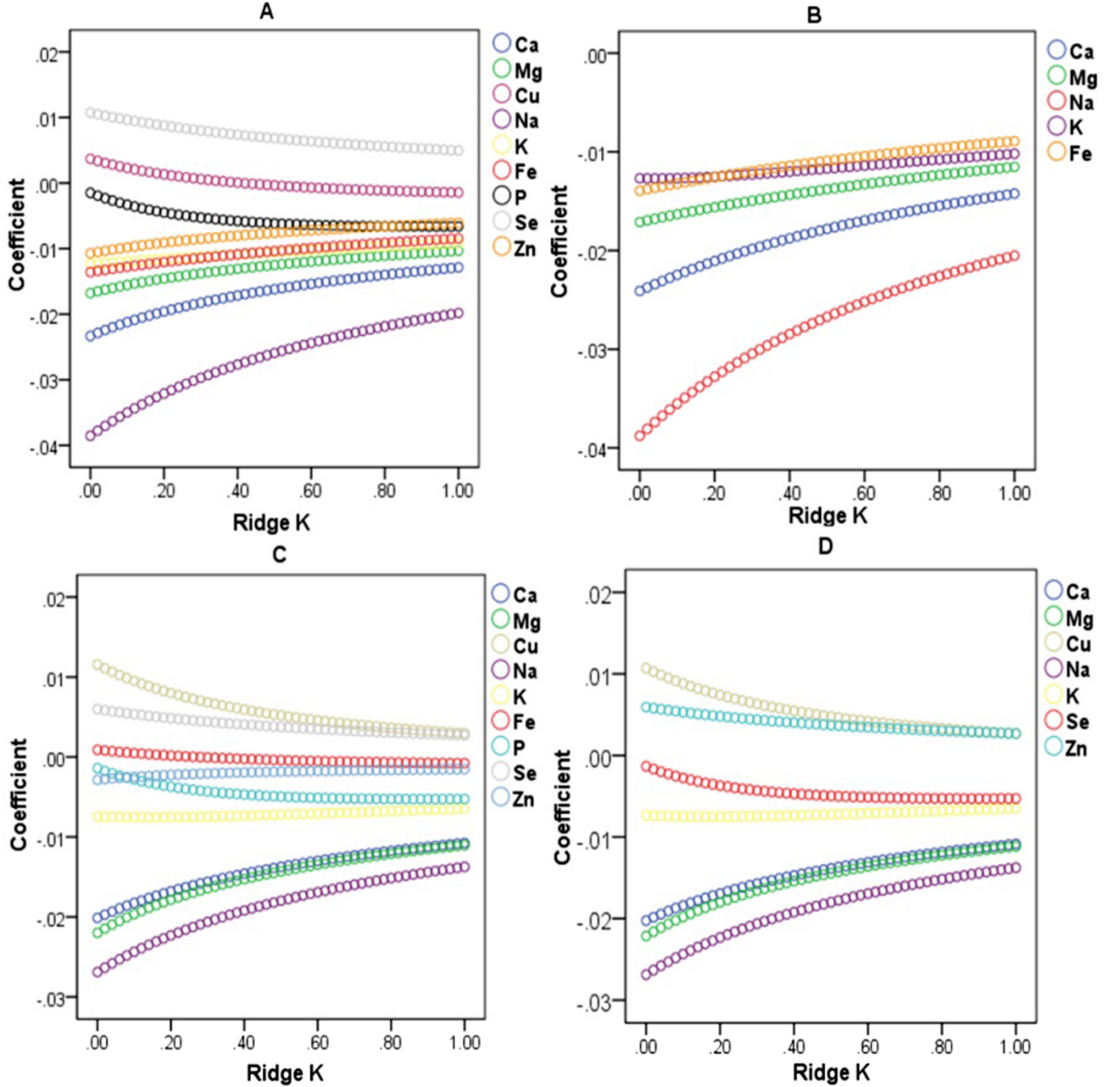
Ridge regression analysis on the relationship between BMI, WC and dietary mineral nutrient intakes. (A) Ridge trace of BMI and dietary mineral intakes. (B) Ridge trace of BMI and the selected dietary mineral intakes after the independent variables with collinearity were eliminated. (C) Ridge trace of WC and dietary mineral intakes. (D) Ridge trace of WC and the selected dietary mineral intakes after the independent variables with collinearity were eliminated.

The collinearity diagnostics showed that the minimum Eigenvalue was 0.009, and the largest Condition Index was 31.417 (greater than 10). There may be collinearity between dietary mineral intakes (Table S2). With increasing ridge *K*, if the absolute value of the ridge regression coefficient of the independent variable changes little and approaches to zero, we need to eliminate these independent variables. When BMI was the dependent variable, the intakes of copper, phosphorus, selenium and zinc were eliminated (Fig. 1A); and WC as the dependent variable, the intakes of iron and phosphorus were eliminated (Fig. 1C). Ridge regression analysis is performed on the selected independent variables. When the coefficient values of the selected independent variables tend to be stable, the coefficients are significantly different from zero, indicating that the influence of these independent variables on the dependent variables is significant (Figs. 1B and 1D).

### Quantile regression analysis of BMI

The results of quantile regression analysis showed that the effects of various mineral intakes on BMI and WC were different at different quantiles. As shown in Table 2, calcium was negatively correlated with BMI (*P* < 0.05 at 0.3–0.8 quantiles). Magnesium was negatively correlated with BMI (*P* < 0.05 at the quantiles of 0.1–0.9). Sodium was positively correlated with BMI (*P* < 0.05 at the 0.2–0.9 quantiles). Potassium was negatively correlated with BMI (*P* < 0.05 at the 0.1–0.9 quantiles). Iron was negatively correlated with BMI (*P* < 0.05 at 0.4–0.5 quantiles). Quantile regression was performed on the excluded independent variables respectively. Copper was negatively correlated with BMI (*P* < 0.05 at 0.3–0.7 quantiles). Phosphorus was positively correlated with BMI (*P* < 0.05 at 0.2–0.3 quantiles). Zinc was negatively correlated with BMI (*P* < 0.05 at 0.6–0.7 quantiles). There were no correlation between selenium intakes and BMI (*P* > 0.05). The correlation between dietary intakes and BMI was getting stronger with the increase of the quantiles.

**Table 2:** Quantile regression coefficient (*P*-value) of dietary mineral intakes and BMI (NHANES, 2007–2014)^a^.

Model^c^	Minerals	Quantiles^b^								
		0.1	0.2	0.3	0.4	0.5	0.6	0.7	0.8	0.9
1	Ca	−0.19 (0.07)	−0.26 (0.06)	−0.38 (<0.01)	−0.50 (<0.01)	−0.66 (<0.01)	−0.70 (<0.01)	−0.71 (<0.01)	−0.76 (<0.01)	−0.69 (0.08)
	Mg	−1.44 (<0.01)	−1.97 (<0.01)	−2.66 (<0.01)	−3.43 (<0.01)	−3.95 (<0.01)	−4.82 (<0.01)	−4.83 (<0.01)	−4.75 (<0.01)	−5.01 (<0.01)
	Na	0.11 (0.22)	0.22 (<0.01)	0.27 (<0.01)	0.33 (<0.01)	0.38 (<0.01)	0.52 (<0.01)	0.60 (<0.01)	0.93 (<0.01)	1.02 (<0.01)
	K	−0.20 (0.03)	−0.21 (0.02)	−0.28 (<0.01)	−0.59 (<0.01)	−0.69 (<0.01)	−0.81 (<0.01)	−0.86 (<0.01)	−0.97 (<0.01)	−1.00 (<0.01)
	Fe	−2.35 (0.66)	−6.26 (0.17)	−9.79 (0.11)	−19.40 (<0.01)	−22.19 (<0.01)	−15.31 (0.11)	−9.76 (0.17)	−14.99 (0.16)	11.22 (0.62)
2	Cu	−0.05 (0.43)	−0.08 (0.15)	−0.24 (<0.01)	−0.25 (<0.01)	−0.33 (<0.01)	−0.37 (<0.01)	−0.30 (0.02)	−0.21 (0.10)	−0.21 (0.05)
3	P	0.35 (0.11)	0.53 (<0.01)	0.43 (0.02)	0.22 (0.34)	0.01 (0.98)	0.01 (0.97)	0.02 (0.95)	0.18 (0.62)	0.55 (0.24)
4	Se	0.27 (0.71)	0.24 (0.76)	0.22 (0.76)	0.20 (0.70)	0.17 (0.81)	0.15 (0.87)	0.11 (0.91)	0.08 (0.95)	0.01 (0.99)
5	Zn	−2.02 (0.77)	−2.63 (0.36)	−3.83 (0.40)	−6.89 (0.23)	−9.24 (0.06)	−12.01 (0.04)	−14.60 (0.02)	−12.83 (0.10)	−12.30 (0.24)

**Notes:**

a Calculated using quantile regression, models adjusted for age, gender, energy.

b Quantile regression coefficient and *P*-value.

c Model 1, Quantile regression analysis of independent variables without collinearity and BMI; Model 2–5, Quantile regression analysis of BMI and collinear independent variables, respectively.

Ca, Calcium; Mg, Magnesium; Cu, Copper; Na, Sodium; K, Potassium; Fe, Iron; P, Phosphorus; Se, Selenium; Zn, Zinc.

### Quantile regression analysis of WC

As shown in Table 3, calcium and magnesium intakes were negatively correlated with WC (*P* < 0.05 at 0.1–0.9 quantiles). Copper was negatively correlated (*P* < 0.05 at 0.2–0.7 and 0.9 quantiles). Sodium was positively correlated with WC (*P* < 0.05 at 0.2–0.9 quantiles). Potassium was negatively correlated with WC (*P* < 0.05). There were no correlation between selenium and Phosphorus intakes and WC (*P* > 0.05). Quantile regression was performed on the excluded independent variables respectively. Iron was negatively correlated with WC (*P* < 0.05 at 0.3–0.4 quantiles). Zinc was negatively correlated with BMI (*P* < 0.05 at 0.3 and 0.6–0.7 quantiles). The correlation between dietary intakes and WC was getting stronger with the increase of the quantiles.

**Table 3:** Quantile regression coefficient (*P*-value) of dietary mineral intakes and WC (NHANES, 2007–2014)^a^.

Model^c^	Minerals	Quantiles^b^								
		0.1	0.2	0.3	0.4	0.5	0.6	0.7	0.8	0.9
1	Ca	−0.84 (0.04)	−1.16 (<0.01)	−1.48 (<0.01)	−1.47 (<0.01)	−1.50 (<0.01)	−1.44 (<0.01)	−1.84 (<0.01)	−2.20 (<0.01)	−1.87 (0.01)
	Mg	−5.28 (<0.01)	−7.52 (<0.01)	−9.22 (<0.01)	−10.12 (<0.01)	−11.79 (<0.01)	−10.58 (<0.01)	−11.97 (<0.01)	−14.21 (<0.01)	−13.51 (<0.01)
	Cu	−0.26 (0.27)	−0.48 (<0.01)	−0.72 (<0.01)	−1.02 (<0.01)	−1.04 (<0.01)	−0.92 (<0.01)	−0.76 (0.01)	−0.64 (0.07)	−0.61 (0.01)
	Na	0.26 (0.34)	0.45 (0.03)	0.65 (<0.01)	0.73 (<0.01)	0.96 (<0.01)	1.08 (<0.01)	1.44 (<0.01)	1.98 (<0.01)	2.26 (<0.01)
	K	−0.66 (0.01)	−1.15 (<0.01)	−1.31 (<0.01)	−1.67 (<0.01)	−2.15 (<0.01)	−1.99 (<0.01)	−2.38 (<0.001)	−2.61 (<0.01)	−2.66 (<0.01)
	P	0.60 (0.33)	0.82 (0.14)	0.33 (0.40)	0.27 (0.61)	0.19 (0.78)	0.02 (0.97)	0.06 (0.93)	0.71 (0.49)	0.18 (0.86)
	Se	0.49 (0.85)	0.42 (0.77)	0.36 (0.81)	0.31 (0.91)	0.25 (0.93)	0.19 (0.93)	0.13 (0.96)	0.04 (0.99)	−0.10 (0.98)
2	Fe	−21.45 (0.40)	−25.69 (0.05)	−41.61 (0.01)	−63.52 (<0.01)	−51.98 (0.05)	−37.25 (0.11)	−16.88 (0.41)	−1.94 (0.95)	24.99 (0.58)
3	Zn	−9.82 (0.50)	−17.73 (0.15)	−31.63 (0.02)	−42.03 (0.05)	−22.45 (0.21)	−23.84 (<0.01)	−31.09 (0.04)	−41.05 (0.05)	−39.55 (0.14)

**Notes:**

a Calculated using quantile regression, models adjusted for age, gender, energy.

b Quantile regression coefficient and *P*-value.

c Model 1, Quantile regression analysis of independent variables without collinearity and WC; Model 2–3, Quantile regression analysis of WC and collinear independent variables, respectively.

Ca, Calcium; Mg, Magnesium; Cu, Copper; Na, Sodium; K, Potassium; Fe, Iron; P, Phosphorus; Se, Selenium; Zn, Zinc.

## Discussion

The study used a large nationally representative sample of adults in the US. It is one of the few studies on the relationship between obesity and mineral intake in American adult diets. Previous studies have demonstrated that overweight or obese individuals exceed their energy needs but do not meet their mineral needs (Astrup & Bügel, 2019). It is important to understand the factors underlying nutritional inadequacies in individuals with overweight or obesity. Our findings further support the effect of mineral nutrients on obesity.

In the adjusted model, after adjusting for energy intake, we found that calcium intake was negatively correlated with BMI and WC at different quantiles, and the association was stronger in overweight and obese individuals. Lower calcium intake was observed among excessive body weight than in normal body weight individuals. The meta analysis found that low dietary calcium intake was a significant risk factor for overweight in adults (Agarwal et al., 2015; Tremblay & Gilbert, 2011). Evidence from randomized clinical intervention trials also suggested that calcium supplementation can lead to weight loss in overweight and obese individuals (Onakpoya et al., 2011). These studies were similar to our findings. In addition, it has been reported that a potentially important role for calcium in the development of diabetes (Isaia, Giorgino & Adami, 2001). High calcium intake can reduce triglyceride accumulation in adipocytes by lowering serum calcium-regulated hormone levels (Major et al., 2008). Calcium can increase the excretion of fat in the feces, thereby reducing body weight (Soares et al., 2012). Hence, our findings suggest that calcium supplementation might play a preventive role.

Similar to our findings, a cross-sectional study found a negative correlation between magnesium intake and obesity or central obesity, lower magnesium intake was observed among excessive body weight (Beydoun et al., 2008). In a randomized controlled trial, the intake addition of 250 mg of magnesium per day to overweight middle-aged women for 8 weeks resulted in weight loss and fat loss (Moslehi et al., 2013). Low magnesium intake may be a risk factor for obesity-related diseases, such as diabetes (Konishi et al., 2017), atherosclerosis (Rosique-Esteban et al., 2018). Other studies have shown that high dietary magnesium intake can be closely related to reduced insulin resistance, which may be particularly beneficial for overweight and obese individuals in the general population (Morais et al., 2017a). Magnesium deficiency contributed to the development of oxidative stress in obese individuals, as this mineral played a role as an antioxidant (Morais et al., 2017b). Magnesium supplementation may appropriately reduce their risk of obesity, but it is still needed to explore the possible mechanism underlying the association.

Recent work indicated that insufficient copper may be important in obesity, ischemic heart disease and metabolic syndrome (Morrell et al., 2017). A cross-sectional study in China showed a strong negative correlation between copper intake and metabolic syndrome, increasing copper intake could reduce the risk of metabolic syndrome, and similar reports in Korea (Choi & Bae, 2013; Qu et al., 2018). These studies were similar to our findings. Serum leptin was positively correlated with serum copper (Olusi et al., 2003). In a mouse model of hereditary copper imbalance, copper have been shown to be an endogenous regulator of lipolysis (Krishnamoorthy et al., 2016). Copper deficiency can significantly increase plasma cholesterol, which can also lead to fat cell hypertrophy and fat accumulation (Yang et al., 2018). Therefore, copper might be used as a dietary supplement.

Our study found that potassium intake was negatively correlated with BMI and WC at different quantiles, lower potassium intake was observed among excessive body weight. However, the cross-sectional study among diverse US Hispanic/Latino adults showed potassium intake was associated with lower BMI and smaller WC in participants (Elfassy et al., 2018). A cross-sectional study in Japan showed that potassium intake was negatively correlated with obesity by estimating dietary intake by urinary potassium (Murakami et al., 2015). In the present study, low potassium intake was a risk factor for hypertension in adults characterized by higher BMI (Stone, Martyn & Weaver, 2016). People who eat more vegetables and fruits had a lower risk of developing metabolic syndrome, while fruits and vegetables were the main source of potassium intake (Aune et al., 2017). A meta-analysis demonstrated a protective effect of adequate potassium intake on obesity and metabolic syndrome (Cai et al., 2016). It is possible that potassium can affect carbohydrate accumulation and glucose homeostasis (Mariosa et al., 2008). Higher potassium intake may be the factor reducing the potential frequency of obesity.

In this study, iron intake was negatively correlated with BMI at the median quantiles and WC at the lower quantiles. A meta-analysis showed that the overweight/obese participants had a significantly increased risk of iron deficiency (Zhao et al., 2015). However, a cross-sectional study in China showed that total and nonheme dietary iron intake was found to be positively associated with obesity (Zhu et al., 2018). This was in contrast to our research, which may be partly due to different ethnic backgrounds, as previous epidemiological studies were conducted in Asia. For biological mechanisms, iron supplementation can maintain high levels of thyroid hormone in plasma to maintain normal metabolic rate, which may be beneficial for weight loss, especially during obese individuals receiving a low-energy diet (Beard, Borel & Peterson, 1997). Future longitudinal studies will help to test whether causal relationship exists between obesity and iron intake.

Our study indicated that selenium intake was not associated with BMI and WC. However, obesity can promote pro-oxidative and pro-inflammatory conditions, potentially increasing the demand for zinc, selenium and other antioxidants (Fernández-Sánchez et al., 2011). Our result showed that zinc intake was negatively correlated with BMI and WC at the higher quantiles. Animal experiments have shown that zinc was associated with obesity and zinc supplementation can reduce body weight and triglyceride levels in rats with high-fat/high-fructose diets (Thoen et al., 2019). It had been shown that zinc deficiency and selenium deficiency can cause oxidative stress in cells (Kieliszek, 2019; Lee, 2018), which may lead to obesity. In the future, we need to study further to explore the relationship between obesity and the intakes of zinc and selenium.

We also found that sodium intake was positively correlated with BMI and WC, sodium intake was higher in obese individuals. When the risk of obesity increased, the effect of mineral intakes would be greater. The relationship between high sodium intake and obesity had been confirmed in present studies. Sodium intake was positively correlated with BMI and body fat in both children and adults, and salt intake can lead to an increase in obesity incidence (Elfassy et al., 2018; He & MacGregor, 2011; Larsen et al., 2013; Ma, He & MacGregor, 2015). Our results were consistent with the above studies. In addition, high-salt diet was an important risk factor for metabolic syndrome and was positively associated with insulin resistance (Oh et al., 2015). In animal models, it had also been demonstrated that a high-salt diet can lead to endogenous fructose production, leptin resistance and excessive appetite, and can lead to obesity, insulin resistance and fatty liver (Lanaspa et al., 2018). In the adjusted model, our result showed phosphorus intake was positively correlated with BMI at the lower quantiles, and was not associated with WC. High phosphorus intake especially from foods processed with phosphate salts can lead to abnormal cellular metabolism and the development of obesity (Anderson, 2013). So we need to explore their real connections and possible mechanisms.

As mentioned earlier, in the QR model, our study showed an association between BMI, WC and mineral intake in American adults. The cross-sectional design of the study is a limitation, because it is difficult to make causal inferences. Therefore, we need further research in the future, and then nutrition education should be conducted in all BMI and WC groups, and the specific mineral intake of individuals in each group should be adjusted to meet their actual needs and weight.

However, there are still some limitations. First, this is a cross-sectional design that limits cause-effect. And there may be the reverse causation, and obesity may lead to increased food intake. In order to assess the longitudinal effect of mineral nutrient intakes on BMI and obesity, more researches are needed. Our future research should focus on the time-effect of the association. In addition, dietary intake data were collected through two 24 h recall surveys, which may be limited by the memory and estimation accuracy of participants, the study may be affected by recall bias. Due to different measurement conditions in different years, there may be measurement bias in this study. Measurement bias and recall bias may not represent the individual’s usual intake. Finally, although we have adjusted for some confounding factors, there may be residual confounding, including some confounding factors not recorded in the data, such as genetic factors. There was also no control of the disease in the participants in the analysis. The data contained diagnosed diseases caused by obesity, such as cardiovascular disease, hypertension, type 2 diabetes and their eating habits may have changed.

## Conclusion

Our study showed that the mineral nutrient intakes were associated with BMI and WC. Nowadays, the incidence of obesity is rising, and adjusting dietary mineral nutrient intakes to develop healthy dietary interventions may help maintain the healthy weight and prevent obesity. However, due to the simultaneous collection of BMI, WC and food intake information, there may be the reverse causation. In order to assess the true relationship between them, we need to do more research.

## Supplemental Information

10.7717/peerj.9127/supp-1 Supplemental Information 1 Raw data.

10.7717/peerj.9127/supp-2 Supplemental Information 2 Tests of Normality based on the Kolmogorov–Smirnova test.

10.7717/peerj.9127/supp-3 Supplemental Information 3 Collinearity Diagnostics.

10.7717/peerj.9127/supp-4 Supplemental Information 4 The distribution of BMI and Waist circumference (density diagram).

## Abbreviations

QR: Quantile Regression
BMI: body mass index
NHANES: the National Health and Nutrition Examination Survey
Ca: Calcium
Mg: Magnesium
Cu: Copper
Na: Sodium
K: Potassium
Fe: Iron
P: Phosphorus
Se: Selenium
Zn: Zinc

## Additional Information and Declarations

### Data Availability

The following information was supplied regarding data availability: The raw data are available as a Supplemental File.
