# Supplementary material for: Association between dietary mineral nutrient intake, body mass index, and waist circumference in U.S. adults using quantile regression analysis NHANES 2007–2014
Source: PeerJ. 2020 May 4;8:e9127. doi: 10.7717/peerj.9127 (PMC7204818; doi:10.7717/peerj.9127)
Supplement: Supplemental Information 2 [file peerj-08-9127-s002.docx]

Supplemental Table 1 Tests of Normality based on the Kolmogorov-Smirnova test

|  | Statistic | df | P |
| --- | --- | --- | --- |
| BMI(kg/m^2^) | 0.070 | 19952 | <0.0001 |
| WC (cm) | 0.037 | 19952 | <0.0001 |
| Age/years | 0.060 | 19952 | <0.0001 |
| Calcium intake(g/d)^a^ | 0.140 | 19952 | <0.0001 |
| Magnesium intake(g/d)^a^ | 0.198 | 19952 | <0.0001 |
| Copper intake(mg/d)^a^ | 0.254 | 19952 | <0.0001 |
| Sodium intake(g/d)^a^ | 0.153 | 19952 | <0.0001 |
| Potassium intake(g/d)^a^ | 0.130 | 19952 | <0.0001 |
| Iron intake intake(g/d)^a^ | 0.196 | 19952 | <0.0001 |
| Phosphorus intake(g/d)^a^ | 0.146 | 19952 | <0.0001 |
| Selenium intake(g/d)^a^ | 0.467 | 19952 | <0.0001 |
| Zinc intake(mg/d)^a^ | 0.277 | 19952 | <0.0001 |

Notes.

^a^ Nutrient intakes were adjusted for energy according to the residual adjustment method.
