# Supplementary material for: Association between dietary mineral nutrient intake, body mass index, and waist circumference in U.S. adults using quantile regression analysis NHANES 2007–2014
Source: PeerJ. 2020 May 4;8:e9127. doi: 10.7717/peerj.9127 (PMC7204818; doi:10.7717/peerj.9127)
Supplement: Supplemental Information 3 [file peerj-08-9127-s003.docx]

Supplemental Table 2 Collinearity Diagnostics^a^

| Model Dimension | Eigenvalue | Condition Index | Variance Proportions | | | | | | | | | |
| --- | --- | --- | --- | --- | --- | --- | --- | --- | --- | --- | --- | --- |
|  |  |  | Constant | Calcium | Magnesium | Copper | Sodium | Potassium | Iron | Phosphorus | Selenium | Zinc |
| 1 | 8.532 | 1.000 | 0.00 | 0.00 | 0.00 | 0.00 | 0.00 | 0.00 | 0.00 | 0.00 | 0.00 | 0.00 |
| 2 | 0.873 | 3.127 | 0.00 | 0.00 | 0.00 | 0.00 | 0.00 | 0.00 | 0.00 | 0.00 | 1.00 | 0.00 |
| 3 | 0.216 | 6.286 | 0.00 | 0.00 | 0.00 | 0.07 | 0.00 | 0.00 | 0.05 | 0.00 | 0.00 | 0.65 |
| 4 | 0.138 | 7.856 | 0.00 | 0.01 | 0.01 | 0.02 | 0.00 | 0.00 | 0.93 | 0.00 | 0.00 | 0.04 |
| 5 | 0.116 | 8.563 | 0.00 | 0.00 | 0.00 | 0.83 | 0.00 | 0.00 | 0.02 | 0.00 | 0.00 | 0.29 |
| 6 | 0.044 | 14.004 | 0.01 | 0.82 | 0.00 | 0.00 | 0.07 | 0.02 | 0.00 | 0.00 | 0.00 | 0.00 |
| 7 | 0.042 | 14.333 | 0.01 | 0.05 | 0.85 | 0.08 | 0.05 | 0.00 | 0.00 | 0.01 | 0.00 | 0.00 |
| 8 | 0.019 | 21.301 | 0.00 | 0.01 | 0.13 | 0.00 | 0.50 | 0.52 | 0.00 | 0.02 | 0.00 | 0.00 |
| 9 | 0.013 | 25.935 | 0.02 | 0.11 | 0.00 | 0.00 | 0.18 | 0.38 | 0.00 | 0.69 | 0.00 | 0.00 |
| 10 | 0.009 | 31.417 | 0.95 | 0.00 | 0.00 | 0.00 | 0.19 | 0.07 | 0.00 | 0.27 | 0.00 | 0.00 |

Notes.

^a^ Dependent Variable: BMI or WC
