# Supplementary figures and images for: Association between dietary mineral nutrient intake, body mass index, and waist circumference in U.S. adults using quantile regression analysis NHANES 2007–2014
Source: PeerJ. 2020 May 4;8:e9127. doi: 10.7717/peerj.9127 (PMC7204818; doi:10.7717/peerj.9127)

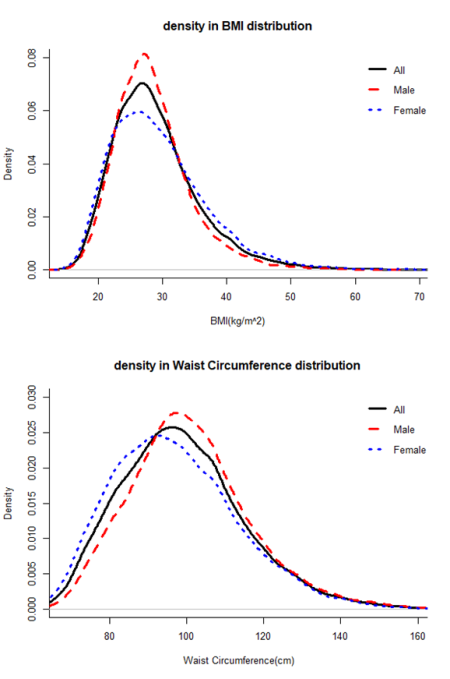

Supplement: Supplemental Information 4 [file peerj-08-9127-s004.png]
